# Distinct Neurotoxicity Profile of Listeriolysin O from *Listeria monocytogenes*

**DOI:** 10.3390/toxins9010034

**Published:** 2017-01-13

**Authors:** Jana Maurer, Sabrina Hupp, Carolin Bischoff, Christina Foertsch, Timothy J. Mitchell, Trinad Chakraborty, Asparouh I. Iliev

**Affiliations:** 1 DFG Membrane/Cytoskeleton Interaction Group, Institute of Pharmacology and Toxicology & Rudolf Virchow Center for Experimental Biomedical Science, University of Würzburg, Versbacherstr. 9, 97078 Würzburg, Germany; jana_maurer@t-online.de (J.M.); sabrina.hupp@ana.unibe.ch (S.H.); carolin_bischoff@gmx.de (C.B.); christina.foertsch@pharmalex.com (C.F.); 2 Institute of Physiology and Pathophysiology, Heidelberg University, Im Neuenheimer Feld 326, 69120 Heidelberg, Germany; 3 Institute of Anatomy, University of Bern, Baltzerstrasse 2, 3012 Bern, Switzerland; 4 Chair of Microbial Infection and Immunity, Institute of Microbiology and Infection, College of Medical and Dental Sciences, University of Birmingham, Edgbaston, Birmingham B15 2TT, UK; t.j.mitchell@bham.ac.uk; 5 Institute for Medical Microbiology, University of Giessen, Schubertstr. 81, 35392 Giessen, Germany; trinad.chakraborty@mikrobio.med.uni-giessen.de

**Keywords:** listeriolysin O, meningitis, acute slices, varicosities, dendritic spines

## Abstract

Cholesterol-dependent cytolysins (CDCs) are protein toxins that originate from Gram-positive bacteria and contribute substantially to their pathogenicity. CDCs bind membrane cholesterol and build prepores and lytic pores. Some effects of the toxins are observed in non-lytic concentrations. Two pathogens, *Streptococcus pneumoniae* and *Listeria monocytogenes*, cause fatal bacterial meningitis, and both produce toxins of the CDC family—pneumolysin and listeriolysin O, respectively. It has been demonstrated that pneumolysin produces dendritic varicosities (dendrite swellings) and dendritic spine collapse in the mouse neocortex, followed by synaptic loss and astrocyte cell shape remodeling without elevated cell death. We utilized primary glial cultures and acute mouse brain slices to examine the neuropathological effects of listeriolysin O and to compare it to pneumolysin with identical hemolytic activity. In cultures, listeriolysin O permeabilized cells slower than pneumolysin did but still initiated non-lytic astrocytic cell shape changes, just as pneumolysin did. In an acute brain slice culture system, listeriolysin O produced dendritic varicosities in an NMDA-dependent manner but failed to cause dendritic spine collapse and cortical astrocyte reorganization. Thus, listeriolysin O demonstrated slower cell permeabilization and milder glial cell remodeling ability than did pneumolysin and lacked dendritic spine collapse capacity but exhibited equivalent dendritic pathology.

## 1. Introduction

Cholesterol-dependent cytolysins (CDCs) represent a broad group of protein toxins that originate from Gram-positive bacteria and contribute substantially to the pathogenicity of their bacterial carriers [1]. Toxins of this group share several common characteristics: their membrane cholesterol-binding capacity is determined by a cholesterol recognition/binding motif (CBM), they have four domains, and they share a highly homologous undecapeptide motif (ECTGLAWEWWR) positioned close to the CBM [2]. They share a common mechanism of pore formation, which involves alignment in oligomeric rings of 30 to 50 monomers along the membrane (prepore), followed by unfolding and the beta-hairpin formation of domain 3 of each monomer to produce an aligned beta-barrel, thereby punching a pore in the membrane. The mechanism of pore formation and membrane penetration by CDCs shares multiple similarities with the proteins of the perforin group, which allows them to be collectively described as the MACPF/CDC (Membrane Attack Complex/Perforin) superfamily of pore-forming proteins [3]. There are some cell effect differences among some toxin members of the group. For example, intermedilysin requires CD59 apart from cholesterol to the bind host cell membrane [4], and listeriolysin O (LLO) is involved in the disruption of intracellular host vacuoles containing listeria [5]. Some differences also exist in their systemic effects, depending on the pathogenic niche that the bacteria occupy. Pneumolysin (PLY) is relevant to the trachea, lungs, inner ear, and brain, where *Streptococcus pneumoniae* (also known as pneumococcus) also prefers to reside [6], and perfringolysin is located in the wounds and muscle tissue infections where *Clostridium perfringens* causes disease [7]. Nevertheless, both toxins are molecularly highly homologous. The pathogenic role of each of them depends on not only the individual molecular properties but also the behavior of the expressing microorganism. 

PLY and LLO are two of the most extensively studied members of the group and share some pathogenic similarities, such as the affinity of their carriers to the brain. *Listeria monocytogenes* and* S. pneumoniae* produce debilitating inflammations of brain meninges, with pneumococcus being considered the leading cause of meningitis in adults [8,9]. The role of PLY in the pathogenesis of meningitis is well known because the elimination of PLY diminishes the severity of pneumococcal diseases in various models [10]. Many non-lytic pathogenic effects of PLY are studied in detail: its synaptotoxic effect [11], astrocyte remodeling capacity [12,13], activation of stress kinases [14], and pro-inflammatory effects [15]. Although most of the effects of PLY depend on its lytic capacity (non-lytic mutants have hardly any pathogenic potential), many of the effects occur at sub-lytic concentrations (concentrations at which most cells survive [13]). Here, a differentiation between lytic capacity (molecular capability to build pores) and lytic effects (pore formation beyond repair accompanied by cell destruction) must be made. 

Much less is known about the neurotoxic potential of LLO, and most information has been obtained from indirect analyses of pathologic brain materials of patients with listerial meningitis and encephalitis [16]. *L. monocytogenes* is an important foodborne pathogen that has a significant impact on both public health and the economy worldwide [17]. Although human infections are rare, *L. monocytogenes* has the potential to cause serious and life-threatening diseases, preferentially in immunocompromised patients, such as septicemia, meningoencephalitis, meningitis, or abortion in pregnant women.

LLO is studied largely in the context of its role in facilitating pathogen internalization, intracellular phagosome disruption, release of *L. monocytogenes* and cell-to-cell spread [18]. LLO is the only member of the CDC group of toxins that is secreted by an intracellular pathogen and thus affects host cell physiology both intra- and extracellularly [19]. Its direct role as a neurotoxin has never been studied. In the case of PLY, for example, a substantial amount of the toxin is released in the cerebrospinal fluid (CSF) during bacterial lysis (approx. 0.1–0.2 µg/mL). For LLO, there is no information regarding its extracellular concentration. Furthermore, reports indicate pH-dependence for LLO with partial loss of activity at physiological pH due to a premature unfolding of the toxin [5]. Thus, despite similarities to other CDCs, LLO differs from them both structurally and functionally. Characterization of its neurotoxic effect, compared to other well-studied neurotoxins of the group such as PLY, represents a question of substantial importance not only for the characterization of the CDCs but also for understanding the pathogenic processes that occur during *L. monocytogenes* meningitis and encephalitis. Thus, we have performed an extensive analysis of LLO neurotoxicity both in primary glial cultures and in acute brain slices, focusing on dendritic and astrocyte changes.

## 2. Results

**Figure 1:**
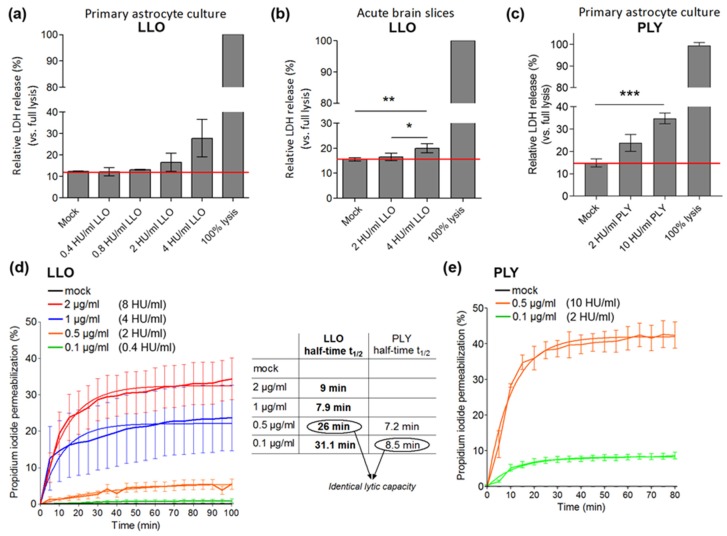
Lytic capacity of listeriolysin O (LLO) in primary glial cells: (**a**) lactate dehydrogenase (LDH) release in primary glial cells after challenge with various concentrations of LLO for 30 min. The red line indicates background LDH release; (**b**) LDH release in acute brain slices, oxygenated with carbogen (95% O_2_/5% CO_2_ mix) after 5 h of LLO exposure; (**c**) LDH release in primary glial cultures after challenge with various concentrations of pneumolysin (PLY) for 30 min. 100% lysis controls were prepared by cell lysis with 1% Triton X-100 in PBS; (**d**) live imaging permeabilization (as judged by propidium iodide nuclear staining) analysis in primary mouse glial cultures after challenge with various amounts of LLO and (**e**) PLY. Total number of cells per field was determined by DAPI nuclear staining at the end of the experiment. Values from non-linear regression analysis of half-times are presented in the table. In (**d**,**e**), toxin concentrations were expressed both as µg/mL and in hemolytic units (HU/mL). All values represent mean ± SEM, *n*= 4–6 independent experiments; * *p* < 0.05, ** *p* < 0.01, *** *p* < 0.001.

First, we examined the cytotoxicity of recombinant LLO. We compared LLO with PLY, a well-studied neurotoxin from the same group. At physiological pH, our recombinant PLY demonstrated hemolytic activity of 20,000 hemolytic units (HU)/mg, whereas the activity of the recombinant LLO was 4000 HU/mg in human erythrocyte lytic test (not shown). In a lactate dehydrogenase (LDH) release assay in primary glial cells, LLO was sub-lytic (defined as non-lytic in brain slices and with less than 10% lysis in primary cells [11,13,20]) at concentrations below 2 HU/mL (Figure 1a,b). PLY was sub-lytic in glial cell cultures at concentrations between 2 and 10 HU/mL (Figure 1c). Live imaging of propidium iodide (PI)-permeable cells demonstrated slower permeabilization kinetics of LLO versus the equally lytic PLY [13] (Figure 1d,e). At concentrations of 2 HU/mL, LLO permeabilized cell slower (half-time of 26 min) compared to PLY (half-time of 8.5 min).

**Figure 2:**
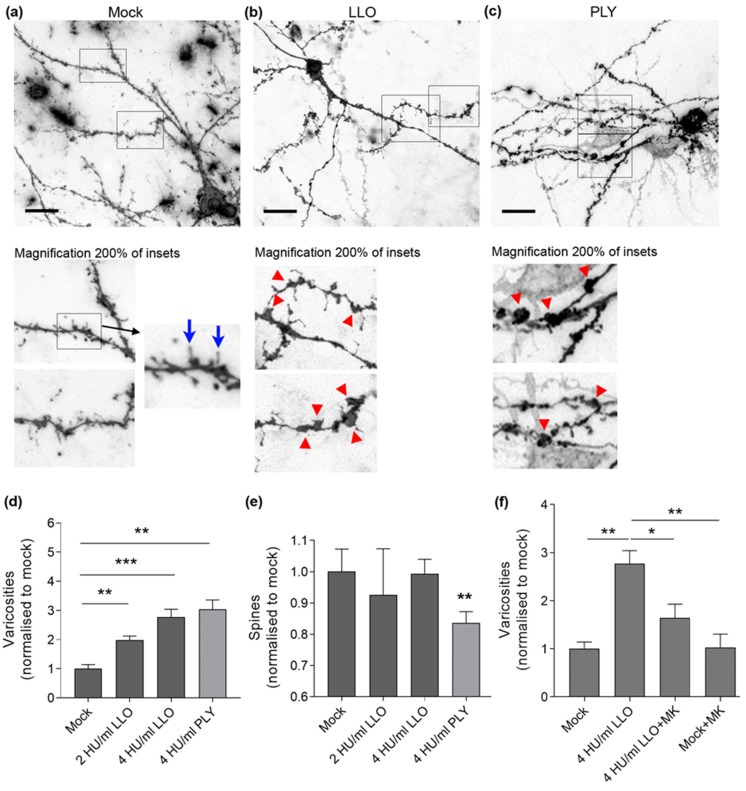
Neurite morphology in acute mouse brain slices after LLO challenge: (**a**) neurons in acute brain slices (PD 10–14) stained with DiI, visualizing the whole neurite tree (dendrites and axons) of intact neurons. In mock-treated samples, normal configuration of dendrites with only accidental widening in the form of varicosity (red arrow) is observed; (**b**) a neuron in the LLO-treated slice (4 HU/mL) with multiple varicosities (red arrows in the magnified fragments of (**b**,**c**)) along dendrites, but preserved dendritic spines (blue arrows in the magnified fragment of (**a**)); (**c**) multiple varicosities and dendritic spine reduction after exposure to 4 HU/mL PLY for 5 h. Scale bars: 20 µm; (**d**) increase in varicosity number (normalized to mock) with increase in the LLO concentration after 5 h exposure, compared with PLY; (**e**) unchanged dendritic spine number (normalized to mock) after exposure to various concentrations of LLO for 5 h. Challenge with 4 HU/mL PLY for 5 h significantly reduces the number of spines; (**f**) partial reversal of the varicosity formation (normalized to mock) by 4 HU/mL LLO after incubation with 10 µM MK801 (NMDA receptor antagonist). All values represent mean ± SEM, *n =* 5 independent experiments; * *p <* 0.05, ** *p <* 0.01, *** *p <* 0.001.

Next, we analyzed the ability of LLO to initiate dendritic and synaptic pathology similar to PLY in an acute brain slice system under continuous oxygenation with carbogen gas (mix of 95% O_2_/5% CO_2_) (see Materials and methods). We prepared acute brain slices from C57Bl/6 mice, postnatal day (PD) 10–14, and incubated them for 5 h with 2 HU/mL and 4 HU/mL LLO. At these concentrations, only varicosity (swellings along the dendrite of neurons) formation increased significantly (Figure 2a–d), while dendritic spines (the structural substrate of synapses) remained unchanged (Figure 2e). Treatment with 4 HU/mL PLY produced both significant increase in varicosity formation (Figure 2d) and decrease in dendritic spines (Figure 2e). 

The formation of varicosities by PLY is glutamate-dependent and can be inhibited by antagonists such as MK801 (non-competitive NMDA glutamate receptor antagonist) [11]. In the case of LLO, varicosity formation was inhibited by 10 µM MK801, which is indicative of the role of glutamate (Figure 2f).

**Figure 3:**
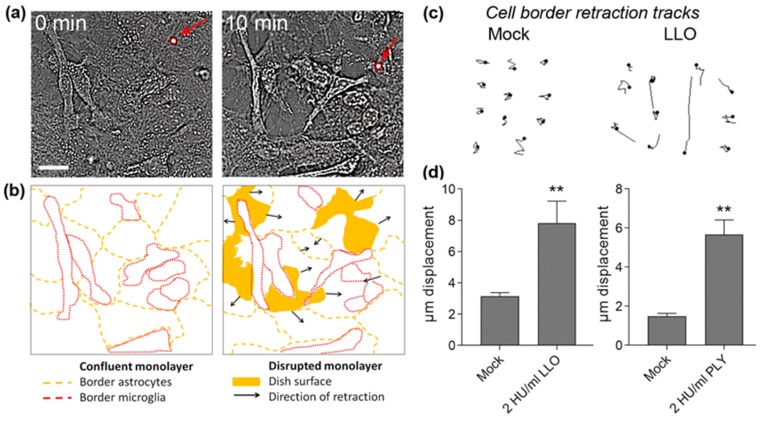
Displacement and cell shape changes of primary astrocytes after LLO exposure: (**a**) transmission images demonstrating a disruption of the glial monolayer (confluent at 0 min) and retraction of non-permeabilized cells (10 min). A PI-labelled cell is visible in the field (red arrow). Scale bars: 20 µm; (**b**) schematic presentation of the cell borders of individual glial cells in (**a**) with outline of the retraction direction and areas of monolayer disruption; (**c**) tracks of cell border displacement of non-permeabilized cells (pooled together; see Materials and Methods for details). Scale bars: 20 µm; (**d**) significant increase of cell border displacement after challenge with 2 HU/mL LLO. Cell border retraction by 2 HU/mL PLY is presented on the right graph. All values represent mean ± SEM, *n =* 40 cells; ** *p <* 0.01.

A major effect of PLY in brains is astrocyte cell shrinkage and shape changes, which can lead to glial remodeling in brain neocortex. LLO was capable of inducing astrocyte cell shrinkage and shape remodeling in non-permeabilized primary astrocytes at concentrations as low as 2 HU/mL (Figure 3a). Membrane permeabilization was judged by PI nuclear staining and non-permeabilized cells remained PI-negative (in Figure 3a, a PI-stained cell with nucleus in red (red arrow)). Shortly after exposure to sub-lytic concentrations of LLO, the confluent glial monolayer in cell culture conditions was disrupted, and the cells retracted (Figure 3a,b). Tracks of cell border displacement of non-permeabilized cells (several pooled together in Figure 3c) demonstrated a significant retraction increase within the first 10 min of LLO exposure (Figure 3d). Similarly, 2 HU/mL PLY produced significant cell border displacement (Figure 3d).

**Figure 4:**
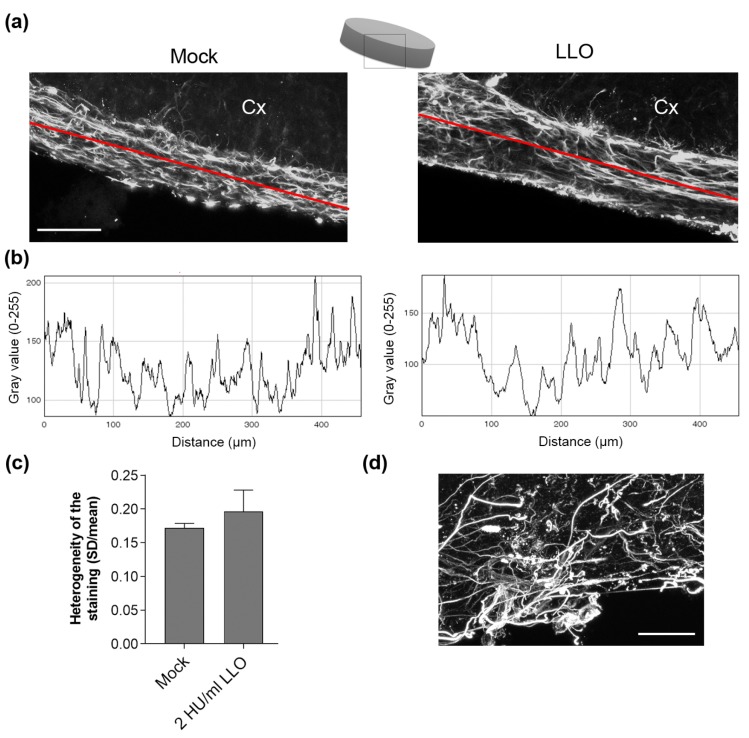
Morphology of the superficial neocortical astrocytes in acute brain slices after LLO: (**a**) immunohistochemistry against GFAP demonstrates a similar morphology and lack of visible astrocyte layer disruption on the surface of the neocortex after 2 HU/mL LLO treatment. Cx indicates the neocortical portion of the slice; (**b**) profile analyses of the immunofluorescent images along the red line in (**a**). Alterations of the homogeneity of distribution and bundling of cells can be determined by heterogeneity analysis (standard deviation (SD) divided by the mean); (**c**) No difference between mock and LLO groups was observed (values represent mean ± SEM, *n =* 5 slices); (**d**) massive control remodeling and disruption of the superficial glial layer by 2 HU/mL PLY. Scale bars: 100 µm.

To translate these findings of astrocyte cell shape changes and retraction to real tissue conditions, we exposed acute brain slices (PD 10–14) to 2 HU/mL LLO for 5 h and analyzed the anti-GFAP (glial fibrillary acidic protein, an astrocyte marker) immunostaining of the astrocytes of *glia limitans* at the neocortical surface, which is considered a barrier against toxic substances from the CSF. Surprisingly, we did not find any apparent disruption of the astrocyte layer or substantial cell shape changes in the slices, despite the evidence of cell shape remodeling in culture (Figure 4a–c). In the similar brain slice culture system, 2 HU/mL PLY initiated strong glial cell remodeling (Figure 4d).

## 3. Discussion

Our experiments demonstrated a distinct neurotoxic profile of LLO that is associated with dendritic varicosity formation in an NMDA-dependent manner and astrocyte cell shape changes in culture. In contrast to PLY, the other typical neurotoxin of the CDC group, LLO demonstrated a delayed cell permeabilization profile, no manifestations of dendritic spine collapse (indicative of diminished synaptotoxic effect), and morphologically significant astrocyte remodeling in brain tissue at equivalent lytic amounts. 

Members of the CDC group have attracted great attention, mostly due to their unique mechanism of membrane penetration, which are observed among members of the MACPF group as well [3]. Cellular analyses have focused primarily on several typical roles of the members, such as the neurotoxicity of PLY [14], and endocytosis, bacterial internalization, and endophagosomalescape by LLO [21,22]. The neurotoxicity of LLO, however, has scarcely been studied, but it carries substantial relevance due to the role of *L. monocytogenes* in juvenile meningitis and encephalitis [16]. 

In our study, LLO demonstrated a permeabilization profile in primary glial cells that was slower than that of PLY with a comparable lytic capacity [13]. Several factors can influence the kinetics of permeabilization, including the (i) binding affinity, oligomerization speed, and pore formation; and (ii) membrane repair and toxin turnover. In the case of LLO, differences in internalization and secondary activation by lower pH are known factors that can be considered partially responsible for these differences [5,23]. Whereas cell permeabilization by CDCs represents a readily observed cell culture phenomenon, cell lysis is a much rarer phenomenon in tissue and organ conditions, and many non-lytic effects may occur [11,12,20]. Disease-relevant concentrations of PLY, for example, are lytic in cultures but not in tissues [24].

It has been shown that at non-lytic concentrations in brain tissue, PLY initiates two major types of neuropathological changes in the neocortex [11]:

- Dendritic varicosity formation (swelling of the dendrites that are mostly NMDA glutamate receptor-dependent). A similar phenomenon is observed in ischemic brain damage [25]. Studies have described the presence of dendritic varicosities as an element of neural damage in models of diabetes and prion disease [26,27].
- Dendritic spine collapse, which represents a morphological substrate of synaptic loss [28]. Dendritic spines represent actin-filled dendritic protrusions, which are very dynamic at the beginning of their formation. A synapse is formed on the top of a dendritic spine, in contact with neighboring neurites. The spine stabilizes and becomes mature [29].

In our study, LLO demonstrated only partial resemblance to PLY; it initiated NMDA-dependent dendritic varicosity formation in a similar manner but surprisingly did not induce the collapse of dendritic spines. The occurrence of dendritic varicosities represents a typical and early pathologic finding in ischemic brain damage and often precedes dendritic spine collapse [25,30]. Apparently, LLO dysregulates glutamate turnover to a sufficient level to cause varicosities but not enough to initiate the collapse of dendritic spines. This did not change, even when we increased the concentration of LLO to lytic levels in tissue. Previous works indicated that dendritic varicosities alone can be reversed to some extent by normalizing the glutamate reuptake by astrocytes or by NMDA receptor inhibition [30]. Although the effects of LLO on dendrites and spines were milder than those of equally lytic concentrations of PLY, we cannot exclude that in combination with other toxic factors from *L. monocytogenes*, this effect would be enhanced [31]. 

LLO is the only member of the CDC toxin group, which is produced by an intracellular pathogen. Nevertheless, some of the toxin is released extracellularly as well. The pathogenic role of intra-/extracellularly released LLO on host cell physiology differs [18]. Intracellular LLO should be rapidly degraded by the host cell and very few should be released in the extracellular fluid. Extracellular toxin released either in the course of massive bacteria lysis and/or killing of host cells, acting on neuronal cell, should be most relevant to our study. The question of whether listerias, once in the central nervous system, reside predominantly intracellularly or proliferate outside host cells is still unclear. Thus, the relevance of our conclusions strongly depends on the intra/extracellular life mode of *L. monocytogenes* in the brain tissue. In contrast to listeria, *S. pneumoniae* releases PLY in the course of meningitis and other relevant pathologies, extracellularly [6].

Listerial meningitis accounts for 8% of all meningitis cases, and its diagnosis represents a major clinical challenge. The pathogen is confirmed in the CSF of only a third of all patients, and in most cases, empirical therapy is applied [8,32]. A specific feature that pathologically differentiates this type of meningitis is the frequent involvement of brain parenchyma (including brain abscesses) and hematogenic dissemination [32]. Evidence for multiple abscesses along interconnected projection areas of the brain implies a possibility for distribution via white matter fiber tracts [33]. LLO is known to be critical for the development of listerial disease: LLO is required to release bacteria from the endosomal compartment in host cells, and toxin deficiency renders bacteria highly non-virulent [34]. At the same time, very high levels of LLO or very early exposure of the pathogen to the immune system may have a deleterious effect on the progress of disease [35]. Apparently, the optimal disease concentration of LLO requires precise adjustment. LLO is a secreted hemolysin, but its concentration in the CSF during listerial meningitis remains unknown. The cellular invasion of listeria into the cortex with subsequent necrotic changes may produce high local toxin concentrations, which cannot be determined by CSF analysis and may not be reflected in the amounts used in the current study [32]. Of particular interest is whether metabolites, such as phospholipids, that are secreted into the extracellular space alter the toxin’s effects.

Another finding of the current work is the ability of LLO, similar to PLY, to initiate non-lytic astrocytic cell shape changes in culture. This indicates that cytoskeletal reorganization and cell shape remodeling represent a feature of other members of the CDC group toxins (apart from PLY) and are associated with early effects on the membrane due to prepore or pore formation by these toxins. In the last years, ample evidence has accumulated that supports the massive cytoskeletal reorganization capacity of PLY at sub-lytic concentrations, which consists of actin remodeling and bundling of microtubules [36,37,38]. Whereas the cell culture remodeling effects of PLY were readily translated in tissues, this was not the case for LLO because, in acute brain slices, LLO failed to remodel astrocytes on the neocortical surface. Various explanations may be given:

- the magnitude and mechanical strength of cell shape changes differ from toxin to toxin;
- the time frame of cell shape changes by LLO may be slower compared to PLY, thus allowing more effective tissue adaptation;
- continuous exposure of tissue to LLO in normal pH conditions may lead to toxin inactivation; or
- inhibitory cellular metabolites specifically inhibit LLO.

In the current study, only the effects of recombinant purified toxins on cells and tissues were analyzed. All bacterial diseases, however, represent complex host/pathogen interactions in which a plethora of bacterial and host defense factors playing pathogenic roles. For example, an obvious difference between *S. pneumoniae* and *L. monocytogenes* is the presence of two distinct phospholipases produced by the latter bacterium that may act in concert with LLO to exacerbate changes in cell function. Furthermore, the phosphatidylcholine-specific phospholipase C from *L. monocytogenes* is an important virulence factor in murine cerebral listeriosis [39]. Our work outlines for the first time the neuropathological effects of LLO, which can contribute to lethality and disability after listerial meningitis. Further work is needed to clarify the crosstalk between LLO and other toxic factors in listerial meningitis. 

## 4. Materials and Methods

### 4.1. LLO and PLY Preparation

LPS-free LLO was expressed and purified from the wild type *L. innocua* 6a strain as described previously [40]. Shortly, overnight bacterial culture grown at 37 °C in BHI broth was used to inoculate the chemically defined minimal medium. Following 48 h incubation at 30 °C, bacteria were removed by centrifugation and the supernatant was concentrated using a Millipore filtration apparatus (Merck Millipore, Billerica, MA, USA) with a cut-off point of 10 kDa. The crude supernatant of LLO was then batch-absorbed for with Q-sepharose or SP-sepharose (Pharmacia, Freiburg, Germany) and pre-equilibrated with loading buffer (50 mM NaH_2_PO_4_, pH 6.2). The non-absorbed fraction was centrifuged and desalted by transferring through a super loop to a HiPrep^TM^26/10 desalting column (Pharmacia, Freiburg, Germany). Loading buffer (50 mM NaH_2_PO_4_, pH 6.2) was used to elute the desalted fraction. This fraction was subsequently filtered through a 0.22 μm filter and loaded onto a Resource S column (GE Healthcare Europe GmbH, Freiburg, Germany) previously equilibrated with 50 mM NaH_2_PO_4_, pH 6.2. The pure toxin eluted reproducibly from the column at 0.21 to 0.28 M NaCl using elution buffer (50 mM NaH_2_PO_4_, 1 M NaCl, pH 5.6). Protein desalting and purification processes were carried out using the high performance chromatography system ÄKTA explorer and UNICORN^TM^ control system (Pharmacia, Freiburg, Germany).

Wild-type PLY was expressed in *Escherichia coli* BL-21 cells (Stratagene, Cambridge, UK) and purified via metal affinity chromatography as described previously [41]. The purified PLY was tested for the presence of contaminating Gram-negative LPS using the colorimetric LAL assay (KQCL-BioWhittaker, Lonza, Basel, Switzerland). All purified proteins showed <0.6 endotoxin units/µg of protein. Hemolytic activity was judged on the basis of standard assay described before [13]. Briefly, one hemolytic unit (HU) was defined as the minimum amount of toxin needed to lyse 90% of 1% human erythrocytes per ml within 1 h at 37 °C.

### 4.2. Cell and Slice Cultures and Culture Treatments

Primary mouse astrocytes were prepared from the cortices of newborn C57BL/6 mice (postnatal day (PD) 3–5) as mixed cultures with microglia in Dulbecco’s modified Eagle’s medium (high glutamate) (Gibco, Thermo Fisher Scientific, Waltham, MA, USA). The growth medium was supplemented with 10% heat-inactivated fetal calf serum (FCS) (PAN Biotech GmbH, Aidenbach, Germany) and 1% penicillin/streptomycin (Gibco). Fourteen days after seeding in 75 cm^2^ cell culture flasks (Sarstedt AG & Co. KG, Nuembrecht, Germany), the cells were harvested. Culture treatment with PLY and LLO was performed in serum-free medium.

Acute brain slices were prepared from infant (PD 10–14) C57Bl/6 mice via decapitation and vibratome sectioning (Vibroslice NVSL, World Precision Instruments, Berlin, Germany) in artificial CSF continuously oxygenized with carbogen gas (95% O_2_, 5% CO_2_) at 4 °C. The slices were allowed to adapt in carbogenated Basal Medium of Eagle (Gibco) with 1% penicillin/streptavidin and 1% glucose at 37 °C for 1 h before being challenged with PLY or with LLO in the 5% CO_2_-buffered medium (pH = 7.3). In these acute slices, cell lysis never exceeded 7% within 12 h.

### 4.3. Live Imaging Experiments

Cells were incubated in Leibowitz medium (Gibco) with stable pH outside a CO_2_-incubator as described in detail previously [13]. Primary mouse glial cultures were visualized on an Olympus Cell^M imaging system (Olympus Deutschland GmbH, Hamburg, Germany) temperized at 37 °C with a combination of heating plate and custom-built microscope incubator with heater and thermostat feedback loop, using 10× and 20× dry objectives. PI and DAPI (4′,6-Diamidin-2-phenylindol) stains (Life Technologies, Thermo Fisher Scientific, Waltham, MA, USA) were used at end concentrations of 1 µg/mL. Cell border displacement measurements were performed by subsequent time-frame analysis of visible light transmission images from Olympus Cell^M imaging system and an ImageJ-based plugin (collection of Tony Collins, MBF “ImageJ for microscopy” collection, https://imagej.nih.gov/ij/plugins/mbf/, section “Particle analysis”, plugin “Manual tracking”). 

### 4.4. Lactate Dehydrogenase (LDH) Test

Lactate dehydrogenase test (CytoTox 96 non-radioactive test; Promega, Madison, WI, USA) was performed according to manufacturer´s instructions. Shortly, LDH released from lysed cells was detected by conversion of a tetrazolium salt into a red formazan product for 30 min with subsequent absorption detection at 560 nm on a Tecan Photometer (Tecan Group AG, Männedorf, Switzerland). 

### 4.5. Neural Staining in Slices and Immunohistochemistry

Acute mouse brain slices were fixed in 1.5% paraformaldehyde (Carl Roth, Karlsruhe, Germany) in PBS (pH = 7.3) for 30 min and processed either for immunohistochemistry or for neuron-specific DiI staining [11,42]. Briefly, crystals of the lipophilic DiI stain (Life Technologies) were positioned on layers IV–VI of the cortex and allowed to diffuse along the membranes of neurons with intact neuritic trees. As only projecting branches with clear morphological characteristics and uninterrupted structure were followed, shorter branched cells positioned close to the crystal (presumably not neurons) were easily avoided.

For the immunohistochemical experiments, acute Vibratome slices were prepared, treated, and fixed in 2% formalin solution. After overnight permeabilization in 1% Triton X-100 in PBS, the slices were blocked in 4% BSA for 1 h and incubated overnight with anti-GFAP rabbit antibody (1:200; Life Technologies) and with secondary antibody goat anti-rabbit tagged with Cy3 (1:200; Dianova GmbH, Hamburg, Germany). All samples were preserved with ProLong antifade reagent (Life Technologies).

### 4.6. Microscopy

All samples were analyzed on an Olympus Cell^M imaging fluorescent system using 10× and 20× dry objective or a 60× oil immersion objective, or on a Leica SP5 laser-scanning microscope (Leica Microsystems Heidelberg GmbH, Mannheim, Germany)/Zeiss LSM 880 with Airyscan (Carl Zeiss AG, Oberkochen, Germany) using 63× oil immersion objectives and optical zoom between 4 and 8. Image processing and analysis were performed using the ImageJ software (version 1.43 for Windows, National Institutes of Health, Bethesda, MD, USA).

### 4.7. Statistical Analysis

Statistical analysis was performed using GraphPad Prism 4.02 for Windows (GraphPad Software Inc., La Jolla, CA, USA). The statistical tests included Mann-Whitney *U*-tests (comparing two groups differing in one parameter) or one-way ANOVA with a Bonferroni post-test (comparing three or more groups differing in one parameter). Permeabilization kinetics analysis was performed by non-linear regression analysis, utilizing one phase exponential association.
